# Spatiotemporal heterogeneity of tumor vasculature during tumor growth and antiangiogenic treatment: MRI assessment using permeability and blood volume parameters

**DOI:** 10.1002/cam4.1624

**Published:** 2018-07-07

**Authors:** Cherry Kim, Ji‐Yeon Suh, Changhoe Heo, Chang Kyung Lee, Woo Hyun Shim, Bum Woo Park, Gyunggoo Cho, Do‐Wan Lee, Dong‐Cheol Woo, Sang‐Yeob Kim, Yun Jae Kim, Dong‐Jun Bae, Jeong Kon Kim

**Affiliations:** ^1^ Department of Radiology Asan Medical Center University of Ulsan College of Medicine Seoul South Korea; ^2^ Asan Institute for Life Sciences University of Ulsan College of Medicine Seoul South Korea; ^3^ Bio‐imaging Research Team Korea Basic Science Institute Chungbuk South Korea; ^4^ Department of Convergence Medicine University of Ulsan College of Medicine and Asan Medical Center Seoul Korea; ^5^ BIOPRISM Co., Ltd. Seoul Korea

**Keywords:** magnetic resonance imaging, spatial heterogeneity, tumor vessels

## Abstract

Tumor heterogeneity is an important concept when assessing intratumoral variety in vascular phenotypes and responses to antiangiogenic treatment. This study explored spatiotemporal heterogeneity of vascular alterations in C6 glioma mice during tumor growth and antiangiogenic treatment on serial MR examinations (days 0, 4, and 7 from initiation of vehicle or multireceptor tyrosine kinase inhibitor administration). Transvascular permeability (TP) was quantified on dynamic‐contrast‐enhanced MRI (DCE‐MRI) using extravascular extracellular agent (Gd‐DOTA); blood volume (BV) was estimated using intravascular T_2_ agent (SPION). With regard to region‐dependent variability in vascular phenotypes, the control group demonstrated higher TP in the tumor center than in the periphery, and greater BV in the tumor periphery than in the center. This distribution pattern became more apparent with tumor growth. Antiangiogenic treatment effect was regionally heterogeneous: in the tumor center, treatment significantly suppressed the increase in TP and decrease in BV (ie, typical temporal change in the control group); in the tumor periphery, treatment‐induced vascular alterations were insignificant and BV remained high. On histopathological examination, the control group showed greater CD31, VEGFR2, Ki67, and NG2 expression in the tumor periphery than in the center. After treatment, CD31 and Ki67 expression was significantly suppressed only in the tumor center, whereas VEGFR2 and α‐caspase 3 expression was decreased and NG2 expression was increased in the entire tumor. These results demonstrate that MRI can reliably depict spatial heterogeneity in tumor vascular phenotypes and antiangiogenic treatment effects. Preserved angiogenic activity (high BV on MRI and high CD31) and proliferation (high Ki67) in the tumor periphery after treatment may provide insights into the mechanism of tumor resistance to antiangiogenic treatment.

## 1 INTRODUCTION

Since neoangiogenesis was identified as a fundamental factor in tumor growth, many antiangiogenic agents have been developed.^1^, ^2^, ^3^, ^4^, ^5^, ^6^ Despite an early expectation that these therapeutic agents would successfully increase survival time in patients with solid tumors, validation of meaningful survival benefits has failed in a considerable number of clinical trials. To explain such unsatisfactory treatment results, a variety of molecular and histologic theories have been suggested. Among these, regional and biophysical heterogeneity of tumor vessels has been recognized as an important mechanism of tumor resistance to treatment. Non‐invasive spatiotemporal analysis of tumor vascular phenotypes is, therefore, required to trace angiogenic alteration during tumor growth or treatment. For this assessment, magnetic resonance imaging (MRI) is useful for providing quantitative information on the spatiotemporal heterogeneity of tumor vasculature over a series of repeated examinations.

Transvascular permeability (TP) and blood volume (BV) are relevant vascular parameters used to characterize tumor vessel phenotype. TP is a concept incorporating plasma‐to‐tissue flux rate, interstitial volume, and perfusion. This parameter can be quantified from a leakage profile of an extracellular fluid contrast agent (CA) (eg, small‐molecular gadolinium [Gd] chelates) on dynamic contrast‐enhanced (DCE) MRI. BV is an intuitive indicator to quantify new vessel formation activity, and is closely related to vessel density and size on histologic examination. In MRI, BV can be measured using intravascular CA (eg, superparamagnetic iron oxide nanoparticles [SPION)). Specifically, SPION‐induced ΔR_2_* on gradient‐echo images is indicative of total BV, and ΔR_2_ on spin‐echo images estimates microvascular BV (ie, diameter <5 μm).^7^

To investigate the spatiotemporal heterogeneity of tumor vasculature, we traced the distribution of TP and BV parameters during tumor growth and antiangiogenic treatment in an orthotopic C6 glioma mice model. In serially acquired MRI examinations with dual injections of extracellular fluid Gd‐DOTA and intravascular SPION, TP and BV parameters were quantitatively estimated in the tumor center, periphery and entire tumor area. Finally, the intratumoral heterogeneity in antiangiogenic treatment effect was interpreted based on time‐ and region‐oriented changes in TP and BV.

## 2 MATERIALS AND METHODS

This study was approved by the Institutional Animal Care and Use Committee of the Asan Institute for Life Sciences (South Korea).

### 2.1 Brain tumor model and study protocol

In 20 male mice (Balb/c, 8‐week old, weight 24‐26 g), C6 glioma cells (1.2 × 10^5^ cells, Korean Cell Line Bank, Seoul, Korea) were injected into the right cerebral hemisphere using a stereotaxic injection kit (Hamilton Co, NV, USA). The holes were generated 1‐mm inferior and 2.5‐mm lateral to the bregma, leaving the dura intact, and the cell‐containing needle tip was advanced 2.2 mm below the dura. Thereafter, cells were injected slowly over a period of 4 minutes.

After tumor cell injection, T_2_‐weighted images (T_2_WIs) were obtained every 3 days to monitor tumor volume change. Day 0 was assigned when the tumor volume reached approximately 2 mm^3^, and MRI examinations were performed on days 0, 4 and 7. The median time interval from cell injection to day 0 was 12 days. On day 0, the animals were randomly divided into control (n = 10) and treatment (n = 10) groups. Mice were treated daily with vehicle (Cremophor EL/95% ethanol [50:50], Sigma‐Aldrich, St Louis, MO, USA) in the control group and sorafenib (orally, 30 mg/kg/d) in the treatment group. Sorafenib is a multireceptor tyrosine kinase inhibitor (TKI), and it has been applied in clinical trials for treating glioblastoma.^8^

### 2.2 MRI examination

For MRI examinations, the mice were positioned on a custom‐made mouse cradle and anesthetized using isoflurane (1.0%‐1.5%) in 70% N_2_O and 30% O_2_. During MRI examination, respiration rate was monitored, and body temperature was maintained to be at 37°C using warm airflow.

All experiments were performed using a 9.4‐T magnet (Agilent 160AS horizontal imaging system; Agilent Technologies, Santa Clara, CA, USA). Prior to intravenous CA injection, T_2_WIs were obtained using the fast‐spin echo sequence (TR/TE, 4000/51 milliseconds; flip angle, 90°; echo train length, 8; slice thickness, 1 mm; number of slice, 12 or 13; field of view (FOV), 20 × 20 mm; matrix, 256 × 256). DCE‐MRI was then performed before and after manual bolus injection of Gd‐DOTA through the tail vein (Dotarem^®^, Guerbet, France [molecular weight, 500 D]; injection dose, 0.1 mL/kg of body weight; injection approximately 30 seconds after scanning start) using the following parameters: TR, 33 milliseconds; TE, 2.8 milliseconds; flip angle, 30°; number of repeated acquisitions, 100; time for each dynamic set, 3.15 seconds; slice thickness = 1; number of slice, 12 or 13; FOV, 20 × 20 mm; matrix, 96 × 96.

Because Gd‐DOTA has a short plasma half‐life (approximately ~ 30 minutes) and a significantly low R_2_ relaxivity (4.17 mmol/L/s) compared with our SPION (40 mmol/L/s),^9^ residual gadolinium after DCE‐MRI examination would not affect SPION‐induced ΔR_2_* and ΔR_2_ measurements.^10^ Thus, SPION‐induced ΔR_2_*and ΔR_2_ were measured after DCE MRI examinations. For measuring BV parameters (ie, ΔR_2_*and ΔR_2_), SPION (30 mg Fe/kg bodyweight) was used as an intravascular susceptibility CA. The pharmacokinetic profile of SPION was detailed in a recent article.^11^ Before and after SPION injection, the R_2_ value was measured using the multiecho spin‐echo sequence (TR, 3000 milliseconds; 15 TEs [10‐150 milliseconds; ΔTE, 10 milliseconds]; flip angle, 90°; slice thickness, 1 mm; number of slice, 12 or 13; FOV, 20 × 20 mm; matrix, 128 × 128), and the R_2_* was measured using the multiecho gradient‐echo sequence (TR, 1500 milliseconds; 12 TEs [5‐60 milliseconds; ΔTE, 5 milliseconds]; flip angle, 30°; slice thickness, 1 mm; number of slice, 12 or 13; FOV, 20 × 20 mm; matrix, 128 × 128), respectively.

### 2.3 Tumor center vs periphery

An experienced radiologist drew three regions‐of‐interest (ROIs) on T_2_WI including the entire tumor, center, and periphery; areas with T_2_ hyperintensity and Gd enhancement were considered tumor tissue. By referring to previous studies that described the active proliferation and angiogenesis in tumor border, the outer one‐third of the tumor area was assigned as periphery and the other area as the center.^12^, ^13^ After drawing the ROI covering the entire tumor areas, the radiologist drew another ROI that passed through two‐thirds of the tumor radius. The inner area of this ROI was considered as the tumor center, and the outer area as the tumor periphery.

### 2.4 Vascular parameters

MRI vascular parameters were measured using Analysis of Functional NeuroImages (AFNI, http://afni.nimh.nih.gov/afni) software. DCE‐MRIs were interpolated to have the same number of matrix and slice number as T_2_WIs. As mentioned above, the FOV, matrix size, slice thickness, and number of slice were the same among T_2_WIs, multiecho spin‐echo, and multiecho gradient‐echo images. Consequently, all MR images had the same geometry. To extract various MR signals from the same anatomic location, MR images were then co‐registered to T2WIs.

Transvascular permeability parameters were quantified using non‐PK‐model‐based analysis of DCE‐MRI. To minimize machine‐ or sequence‐induced variations, the time‐intensity curve was normalized by dividing the signal intensity at each time point with the mean pre‐contrast signal intensity. Thereafter, the maximum enhancement rate (MaxEnh) (maximum signal intensity/mean pre‐contrast signal intensity) and initial area under the curve (IAUC) over 60 seconds were measured.

For measuring BV parameters, pre‐ and post‐SPION R_2_* and R_2_ values were calculated according the following equation:

(1) $$\text{SI}=M_{0}\cdot e^{-R_{2}\left({}^{*}\right)\cdot t}$$

where SI is the MR signal intensity, M_0_ is the proton density, *t* is the echo time, and R_2_ (*) is the transverse relaxation rate (ie, R_2_ or R_2_*). The relative BV weighted for macrovascular and microvascular blood vessels was determined as SPION‐induced ΔR_2_* and ΔR_2_, respectively.^14^, ^15^, ^16^

To evaluate the regional variation of tumor vascular phenotypes, the center‐to‐periphery ratio of each vascular parameter was calculated (eg, MaxEnh _center/periphery_ = MaxEnh_center_/MaxEnh_periphery_). The time‐dependent changes in tumor volume and vascular parameters were estimated with the ratio between examination days (eg, MaxEnh_day4/day0_ =MaxEnh_day4_/MaxEnh_day0_).

### 2.5 Histopathologic examination

Immediately after the final MRI examination, the brains of the mice were removed and embedded in paraffin blocks. Cell density was measured using hematoxylin‐eosin staining. Angiogenic activity was estimated using immunohistochemical staining for CD31 and vascular endothelial growth factor receptor 2 (VEGFR2), as described in a literature.^17^ The degree of cell proliferation and apoptosis were measured using Ki67 and α‐caspase 3 staining methods, respectively. In addition, the amount of pericyte was assessed by NG2 staining. In the mid‐coronal section of formalin‐fixed glioma, brain sections were stained with CD31 (Abcam, 1:50), VEGFR2 (Cell Signaling, 1:50), Ki67 (Thermo Fisher, 1:100), and α‐caspase 3 (Cell Signalling, 1:4000) using an automated slide preparation system Benchmark XT (Ventana Medical Systems Inc, Tucson, AZ, USA). The positive signals were amplified using ultraView Universal DAB detection kit (Ventana Medical Systems Inc.), whereas the sections were counterstained with hematoxylin reagent. NG2 staining (Abcam, 1:500) was performed using immunofluorescence multiplex system accomplished with PerkinElmer Opal kit (Perkin‐Elmer, Waltham, MA). For NG2 staining, positive signals were visualized using Opal 520 TSA plus (1:3000) and scanned using the Vectra 3.0 Automated Quantitative Pathology Imaging System (Perkin‐Elmer, Waltham, MA).

Similar to the MRI analysis, each tumor section was divided into the tumor center and periphery. Thereafter, the number of positive signals in the tumor ROI was analyzed on digitized TIF images using ImageJ software (National Institutes of Health, Bethesda, MD, USA). The cells on hematoxylin‐eosin staining and positive signals from CD31, and VEGFR2 staining were automatically segmented according to thresholds of red‐green‐blue color values. For NG2 and α‐caspase 3 staining, the positive signal was segmented by thresholds of hue‐saturation‐brightness values. Then, the fraction of positive signal area (ie, %area) was measured using the particle analyzer function in ImageJ.

### 2.6 Statistical analysis

All statistical analysis was performed using commercially available software (PRISM version 5.01, GraphPad Inc, La Jolla, CA USA). In all parameters, both inter‐ and intra‐group data were compared using non‐parametric methods, including the Wilcoxon rank sum test for two paired observations, the Mann‐Whitney test for two unpaired observations, and the Friedman test for three paired observations. When there was a significant difference in the Friedman test, a post‐hoc analysis was performed using the Dunn test. Differences with *P* < .05 were considered to be statistically significant.

## 3 RESULTS

**Figure 1:**
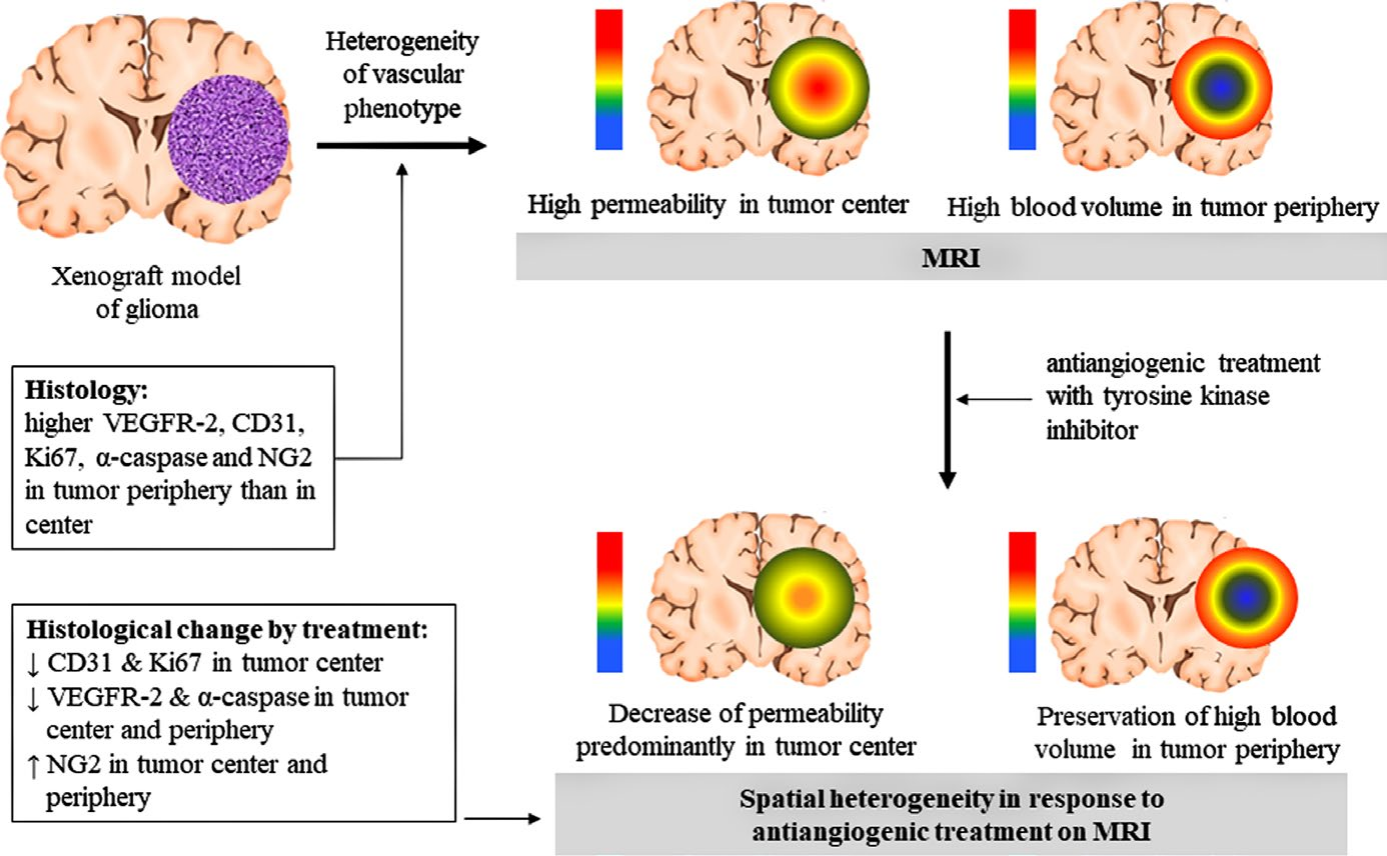
Summary of study results. Magnetic resonance imaging depicts spatial heterogeneity of tumor vascular phenotypes as high transvascular permeability in the tumor center, whereas blood volume was high in the periphery. This pattern became more apparent with tumor growth. The response to antiangiogenic treatment (tyrosine kinase inhibitor) was also heterogeneous according to tumor region, as permeability was predominantly decreased in the tumor center, whereas high blood volume in the periphery was preserved. This MRI findings can be histologically interpreted as treatment‐induced vascular normalization (ie, decreased apoptosis and increased pericyte volume) in the tumor center, whereas high VEGFR2, CD31, and NG2 in tumor periphery support MRI findings of high blood volume in the tumor periphery

**Figure 2:**
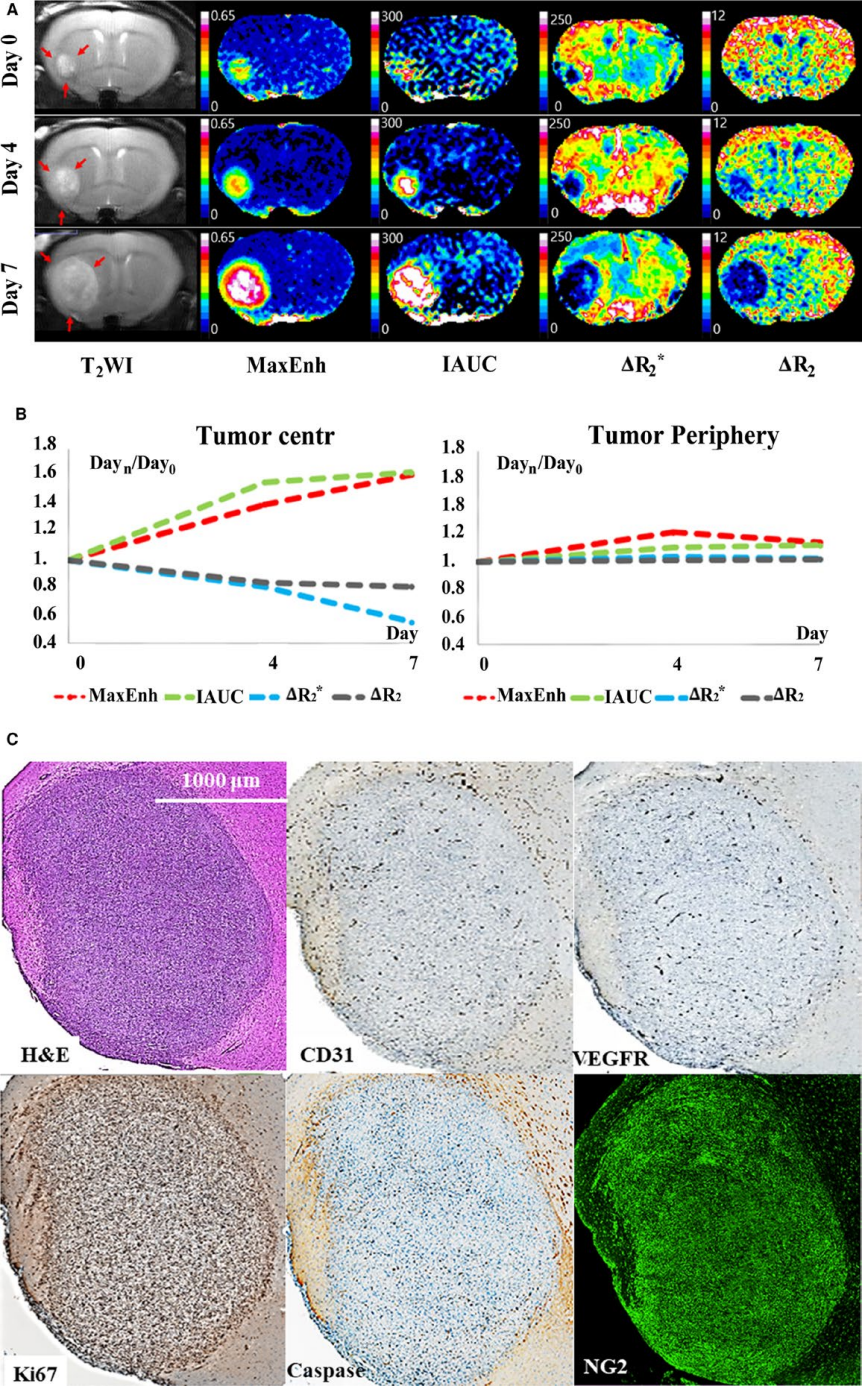
Magnetic resonance imaging and histopathology results in a representative control mouse. A, T_2_‐weighted imaging (T_2_ WI) and vascular parameters. T_2_ WI demonstrated tumor growth from day 0 to day 7. Color maps of vascular parameters demonstrate apparent spatial heterogeneity in the distribution of vascular parameters. Maximum enhancement rate (MaxEnh) and initial area under the curve (IAUC) (ie, indicators of transvascular permeability) are higher in the tumor center than in the tumor periphery. In contrast, ΔR_2_* and ΔR_2_ (ie, indicators of blood volume) are higher in the tumor periphery than in the tumor center. This spatial heterogeneity of vascular phenotypes becomes stronger from day 0 to day 7. B, Plots of time‐dependent relative changes in vascular parameters (ie, parameter_day n/day 0_). MaxEnh and IAUC increased in the tumor center, but not in the periphery. ΔR_2_* and ΔR_2_ tended to decrease in the tumor center, but maintained at initial value in the periphery. C, On histopathological examination, the tumor periphery exhibits higher expression of CD31, VEGFR‐2, and Ki67 than the tumor center, thereby indicating spatial heterogeneity in the activity of angiogenesis and cell proliferation

**Figure 3:**
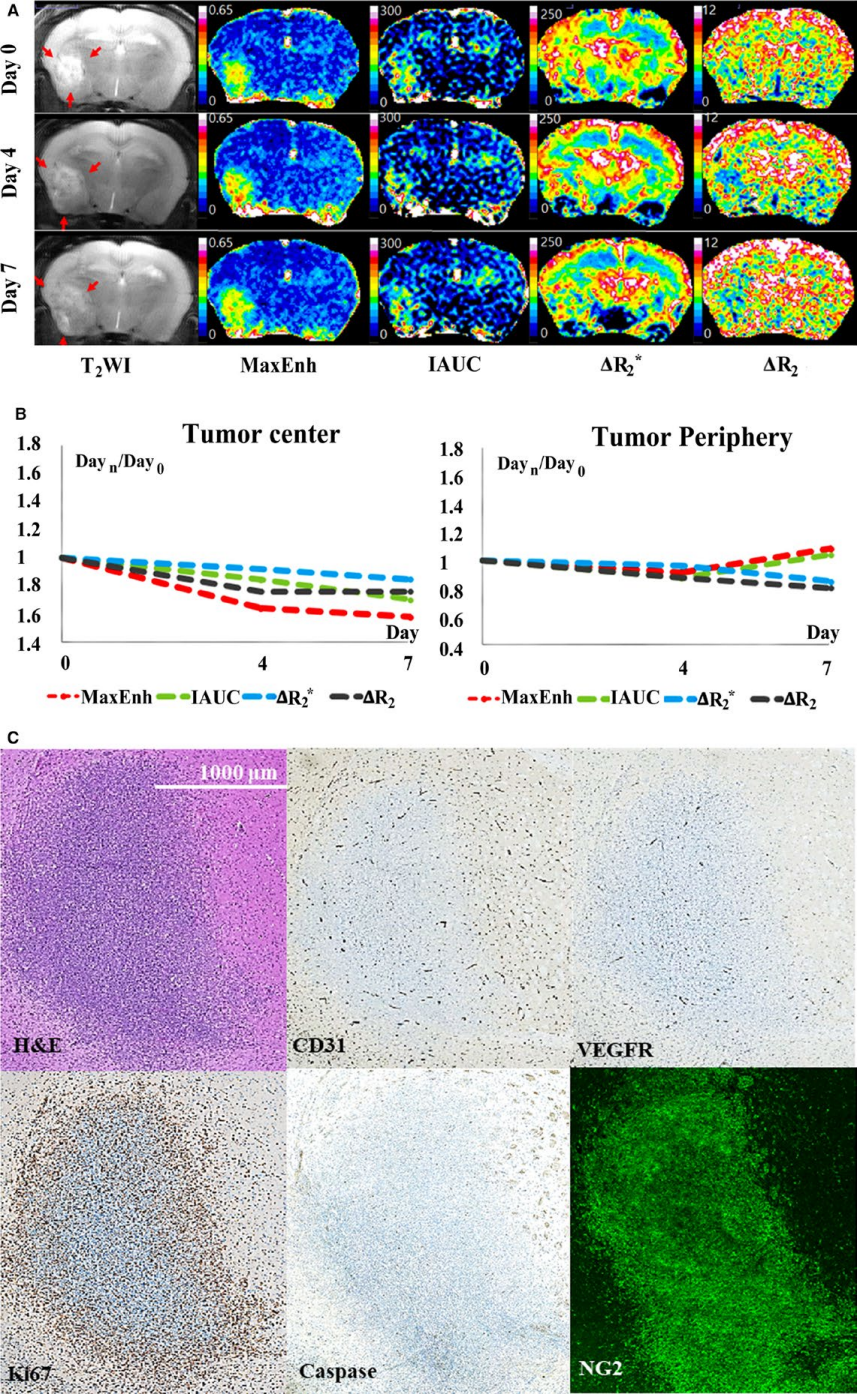
Magnetic resonance imaging and histopathology results in a representative treatment mouse. A, T_2_‐weighted imaging (T_2_ WI) and vascular parameters. T_2_ WI demonstrates treatment‐induced suppression of tumor growth, compared with control mice (see Figure 1A). Color maps of vascular parameters demonstrate apparent spatial heterogeneity in treatment effect within the tumor. Significant treatment‐induced vascular alterations are identified predominantly in the tumor center. Compared with the control group, in the tumor center, antiangiogenic treatment significantly suppressed the increase in transvascular permeability (maximum enhancement rate [MaxEnh] and initial area under the curve [IAUC]) and the decrease in blood volume (ΔR_2_* and ΔR_2_) during the follow‐up period, which are typical temporal changes in control mice (see Figure 1A,B). B, Plots of time‐dependent relative changes in vascular parameters (ie, Parameter_day n/day 0_). Due to significant antiangiogenic treatment effect in the tumor center, treatment mouse exhibits significantly lower MaxEnh and IAUC and higher ΔR_2_* in the tumor center than the control mouse (see Figure 2A,B). In contrast to the tumor center, the tumor periphery exhibits no obvious difference between control and treatment mice. In particular, high ΔR_2_* and ΔR_2_ maintained at the initial value in tumor periphery during follow‐up period. C, CD31 and Ki67 staining demonstrates spatial heterogeneity in the effect of antiangiogenic treatment, as their expression is suppressed predominantly in the tumor center, whereas the tumor periphery preserves active expression (see Figure 1C). VEGFR2 expression is apparently decreased in both the tumor center and periphery compared with a control mouse (see Figure 2C)

Study results are summarized in Figure 1, and representative control and treatment mice are presented in Figures 2 and 3.

### 3.1 Tumor volume

**Figure 4:**
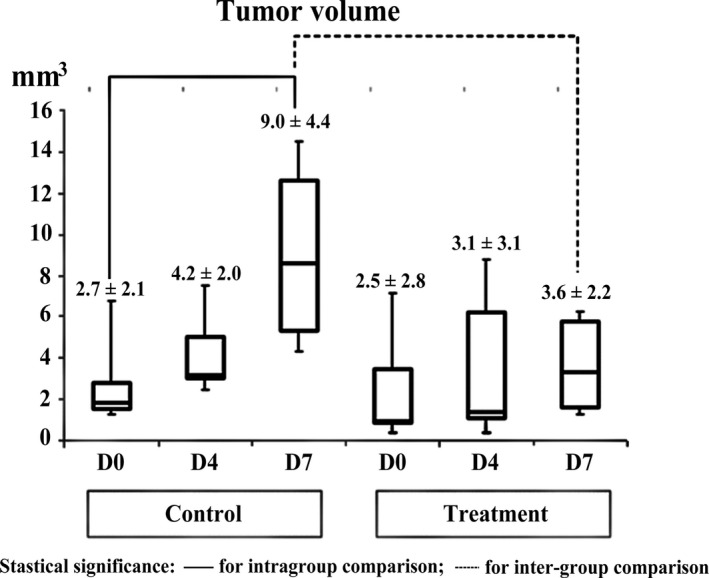
Comparison of tumor volume change between control and treatment mice. Although tumor volume on day 0 was similar between the two groups, the control group revealed statistically significant tumor growth from day 0 to day 7. Accordingly, the control group showed significantly greater tumor volume than the treatment group on day 7. Statistical significance was marked with solid line for intra‐group comparison and with dotted line for intergroup comparison

Time‐dependent changes in tumor volume are summarized in Figure 4. Tumor volume on day 0 was similar between the control and treatment groups (*P* > .05). The control group exhibited statistically significant tumor growth between day 0 and day 7 (volume_day7/day0_ = 3.9 ± 1.8, *P* = .01). The treatment group showed no significant volume change during the follow‐up period (*P* > .05), thereby validating successful suppression of tumor growth by antiangiogenic treatment. Accordingly, the control group showed significantly greater tumor volume than the treatment group on day 7 (*P* < .01).

### 3.2 Spatial heterogeneity of tumor vasculature on initial MRI

**Figure 5:**
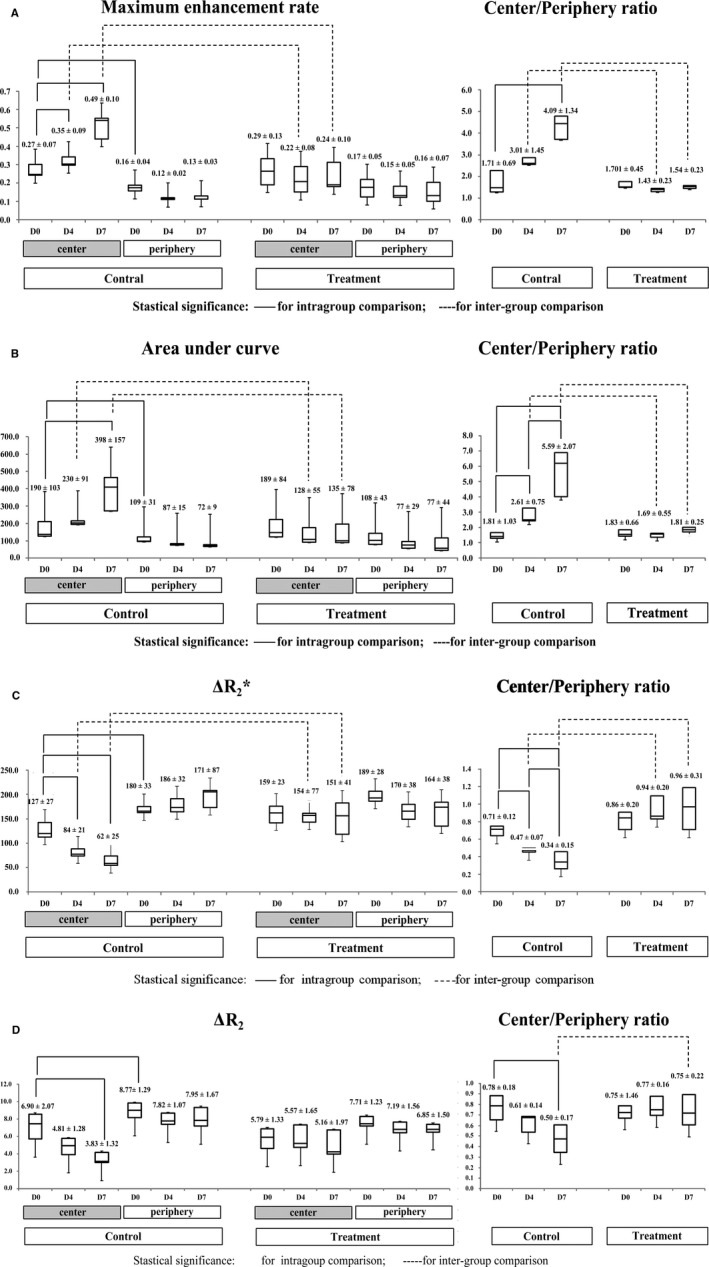
Spatiotemporal analysis of tumor vascular phenotypes. Statistically significant differences are indicated by solid lines (intra‐group comparisons) and dashed lines (intergroup comparisons). A and B, Temporal changes and center‐to‐periphery ratios of maximum enhancement rate (MaxEnh) (A) and initial area under the curve (IAUC) (B). The initial magnetic resonance image day 0 revealed higher MaxEnh and IAUC in the tumor center than in the periphery. With tumor growth in the control group, both parameters increased in the tumor center and similarly maintained in the tumor periphery, thereby demonstrating tendencies toward increasing center‐to‐periphery ratios. Antiangiogenic treatment suppressed such temporal changes only in the tumor center but not in the periphery. Consequently, the treatment group exhibits lower MaxEnh and IAUC in the tumor center than in the control group. C and D, Temporal changes and center‐to‐periphery ratios of ΔR_2_* (C) and ΔR_2_ (D). On day 0, both groups demonstrated higher ΔR_2_* and ΔR_2_ in the tumor periphery than in the center. With tumor growth in the control group, both parameters demonstrated a tendency toward decrease in the tumor center from day 0 to day 7, whereas being similar in the tumor periphery. These changes demonstrated a strong tendency toward decreasing ΔR_2_*_center/periphery_ with tumor growth. Antiangiogenic treatment suppressed such temporal changes in control mice. Consequently, the treatment group demonstrated higher center‐to‐periphery ratios of ΔR_2_*(day 4 and 7) and ΔR_2_ (day 7) than in the control group

In all mice, the initial MRI on day 0 demonstrated significant spatial heterogeneity in the distribution of TP and BV while all vascular parameters were similar between the control and treatment groups (*P* > .05 in all intergroup comparisons). On this MRI, TP parameters (MaxEnh and IAUC) were significantly greater in the tumor center than in the periphery (center‐to‐periphery ratio for TP parameters, 1.70‐1.83; *P* < .05 for both parameters) (Figure 5A,B). In contrast, BV parameters (ΔR_2_* and ΔR_2_) were apparently greater in the tumor periphery than in center (center‐to‐periphery ratio for BV parameters, 0.71‐1.86; *P* < .05 for both parameters) (Figure 5C,D).

### 3.3 Heterogeneous vascular changes during tumor growth

As shown in Figure 5, in the control group, spatial distribution pattern of TP and BV (ie, prominent MaxEnh and IAUC in the tumor center, and high ΔR_2_* and ΔR_2_ in tumor periphery) became more apparent as the tumor grew. This tendency can be clearly described by increasing center/periphery ratio of TP parameters (MaxEnh_center/periphery_: 1.71 on day 0 to 4.09 on day 7; IAUC_center/periphery_: 1.81 on day 0 to 5.59 on day 7) and decreasing center/periphery ratio of BV parameters (ΔR_2_* _center/periphery_: 0.71 on day 0 to 0.34 on day 7; ΔR_2 center/periphery_: 0.78 on day 0 to 0.50 on day 7).

MaxEnh was greater on days 4 and 7 than on day 0 in the tumor center (MaxEnh_day4/0_ = 1.28 ± 0.17; MaxEnh_day7/0_ = 1.90 ± 0.64; *P* < .05 for both comparisons), but were similar in the periphery (*P* > .05 for all intra‐group comparisons) (Figure 5A). IAUC in the tumor center was significantly greater on day 7 than on day 0 (IAUC_day7/0_ = 2.40 ± 1.11; *P* = .04), whereas IAUC in the tumor periphery exhibited no significant change during the follow‐up period (*P* > .05) (Figure 5B). In accordance with such different temporal changes between tumor regions, the MaxEnh_center/periphery_ was greater on day 7 than on day 0 (*P* = .03), and IAUC_center/periphery_gradually increased in every time window (*P* < .05 for all intra‐group comparisons) (Figure 5A,B).

Blood volume parameters in the control mice demonstrated a tendency toward decrease in the tumor center according to tumor growth, whereas tumor periphery maintained high BV on three MRIs (Figures 2 and 5C,D). ΔR_2_* in tumor center significantly decreased from day 0 to days 4 and 7 (ΔR_2_*_day4/0_ = 0.68 ± 0.20; ΔR_2_*_day7/0_ = 0.50 ± 0.21; *P* < .05 for both comparisons). ΔR_2_ in the tumor center also reduced from day 0 to day 7 (ΔR_2 day7/0_ = 0.59 ± 0.23; *P* = .02). However, both ΔR_2_* and ΔR_2_ in tumor periphery exhibited no significant change during the follow‐up period (*P* > .05). Consequently, the control mice demonstrated a strong tendency toward decreasing ΔR_2_*_center/periphery_ in every time window (*P* < .05 for all intragroup comparisons).ΔR_2__center/periphery_ was significantly decreased from day 0 to day 7 (*P* = .04).

### 3.4 Spatial heterogeneity in antiangiogenic treatment effect

The effect of TKI treatment on MRI was spatially heterogeneous within each tumor as significant treatment‐induced angiogenic alterations were identified predominantly in the tumor center (Figure 3). In the tumor center, TKI treatment significantly suppressed increases in TP parameters, and a decrease in BV parameters (ie, typical change pattern in control mice), thereby revealing similar values during the follow‐up period (Figure 4). Accordingly, on days 4 and 7, the treatment group exhibited significantly lower MaxEnh (0.22 vs 0.35 on day 4; 0.24 vs 0.49 on day 7), lower IAUC (128 vs 230 on day 4; 135 vs 398 on day 7), and higher ΔR_2_* (154 vs 84 on day 4; 151 vs 62 on day 7) in the tumor center than the control group (*P* < .01 for all intergroup comparisons). The ΔR_2_ also exhibited no temporal change in tumor center in the treatment group (*P* > .05) although such effect did not yield an intergroup difference.

In contrast to the tumor center, the tumor periphery demonstrated no obvious treatment effect on TP and BV; no statistical significance was noted via inter‐ and intra‐group comparisons (*P* > .05). In particular, high ΔR_2_* and ΔR_2_ values were maintained in the tumor periphery during the follow‐up period (Figure 3).

Due to different temporal changes of vascular parameters in the tumor center between the control and treatment groups, TP_center/periphery_ and BV_center/periphery_ were also clearly different between the two groups (Figure 5). The treatment group demonstrated significantly lower MaxEnh _center/periphery_ and IAUC _center/periphery_, compared with the control group, on days 4 and 7 (*P* < .01). ΔR_2_* _center/periphery_ was higher in the treatment group than in the control group on both days 4 and 7 (*P* < .01). ΔR_2 center/periphery_ was also higher in the treatment group on day 7 (*P* = .02).

### 3.5 Analysis of the entire tumor area

In contrast to region‐oriented analysis, inter‐ and intra‐group comparisons of the entire tumor area revealed no statistically significant differences (*P* > .05 in all intra‐ and intergroup comparisons).

### 3.6 Histopathological results

**Figure 6:**
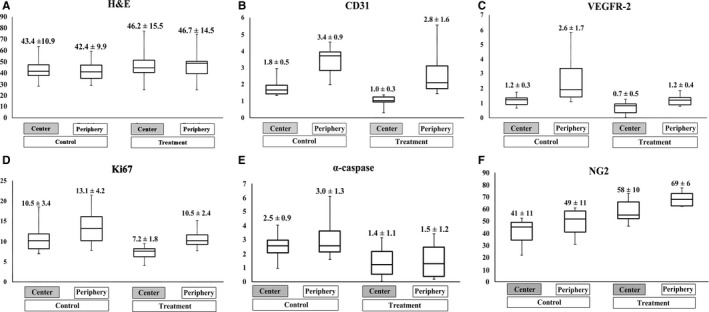
Spatial analysis of histologic study. Box‐and‐Whisker graphs demonstrate the region‐oriented comparison of cell density on hematoxylin‐eosin staining (A), vascular density on CD31 staining (B), angiogenic activity on VEGFR2 staining (C), proliferation activity on Ki67 staining (D), apoptosis on α‐caspase 3 staining (E), and amount of pericyte on NG2 staining (F). The control group demonstrated higher expression of CD31, VEGFR2, Ki67, and NG2 in the tumor periphery than in the center, whereas the cell density was not spatially different. Antiangiogenic treatment yielded significant suppression of CD31 and Ki67, predominantly in the tumor center, whereas the tumor periphery showed no significant treatment effect. VEGFR2 and α‐caspase 3 expression demonstrated apparent treatment‐induced decrease in both the tumor center and periphery. NG2 expression exhibited significant treatment‐driven increase in both the tumor center and periphery

Representative histopathological findings are presented in Figures 2 and 3; the region‐oriented comparison between the control and treatment groups is summarized in Figure 6. The control group demonstrated higher expression of CD31 (3.4 vs 1.8), VEGFR2 (2.5 vs 1.2), Ki67 (13.4 vs 10.5), and NG2 (49 vs 41) in the tumor periphery than in the center, whereas cell density (ie, % area of cells) was not spatially different on hematoxylin and eosin staining. TKI treatment yielded significant suppression of CD31 and Ki67, predominantly in the tumor center, as their % area was lower in the treatment group than in the control group (*P* < .01). However, in the tumor periphery, the TKI treatment effect on these histopathological parameters was insignificant as they were similar between the two groups (*P* > .05). VEGFR2 and α‐caspase 3 expression demonstrated apparent treatment‐induced decrease in both the tumor center and periphery (*P* < .05), and NG2 expression exhibited significant treatment‐driven increase in both the tumor center and periphery (*P* < .01).

## 4 DISCUSSION

Heterogeneity of the tumor microenvironment has emerged as a critical concept for understanding the mechanisms of disease progression and evasion from treatment. Accordingly, the intratumoral diversity of vascular phenotypes is an important characteristic now recognized to affect antiangiogenic treatment, and has been intensively investigated in preclinical and clinical studies.^18^, ^19^, ^20^, ^21^, ^22^, ^23^ As an extension of these efforts, this study assessed region‐ and time‐dependent changes in TP and BV in MRI with dual injection of intravascular and extracellular fluid CAs.

The most important finding of this study was the spatiotemporal heterogeneity in both vascular phenotypes and the treatment responses of glioma. In the control group, TP‐dependent Gd‐DOTA accumulation (ie, MaxEnh and IAUC) was greater in the tumor center than in the periphery, and BV‐dependent contrast effect of SPION (ie, ΔR_2_* and ΔR_2_) was dominant in the tumor periphery. This region‐dependent distribution of vascular parameters became more apparent with tumor growth, as clearly reflected by increasing TP_center/periphery_ and decreasing BV_center/periphery_ during the follow‐up period. These MRI results can be biologically interpreted as upregulated, less‐leaky vessel formation in the tumor periphery, and elevated plasma‐to‐interstitium flux in the tumor center.

The high BV and low TP in the tumor periphery during tumor growth may be explained by neoangiogenesis and vascular stabilization. It has been established that the tumor periphery exhibits highly active VEGF‐associated angiogenesis, which increases the number and size of vessels by accelerating endothelial cell differentiation/proliferation, primitive vessel generation, vasodilation, and vessel lengthening.^24^, ^25^, ^26^, ^27^ In our histopathological examinations, these processes manifested as high VEGFR2 and CD31 expression in the tumor periphery. In addition, the tumor periphery exhibits enhanced vascular stabilization under mediation of various growth factors such as PDGF, angiopoietins‐1, and tumor growth factor‐β. Newly generated primitive vessels are thus matured and covered by supporting matrix and cells,^28^ thereby suppressing uncontrolled vascular leakiness. Such vascular stabilization was also presented as increased NG2 activity (ie, pericyte amount) in the tumor periphery in our study. Therefore, our results of high BV and low TP in the tumor periphery are evidential MRI reflections of new vessel generation and maturation that constitute the invasive margin of the tumor.

Compared with the tumor periphery, the tumor center demonstrated higher TP and lower BV. These findings may be associated with vascular degeneration and destabilization. These vascular alterations―known as “vascular regression”―are characterized by progressive disengagement of endothelial cells from surrounding supportive structures in the absence of angiogenic stimulation.^29^, ^30^, ^31^ This phenomenon is primarily led by Ang‐2, which induces endothelial cell apoptosis and detachment of endothelial cells from surrounding pericytes.^24^, ^25^, ^30^, ^31^ Our histologic study also showed relatively low expression of CD31, VEGFR2, and NG2 in the tumor center in comparison with tumor periphery. In this sense, decreased BV and elevated TP in the tumor center are relevant MRI presentations of vascular regression.

As expected from the vascular heterogeneity in the control group, the effect of antiangiogenic treatment also led to apparent regional variations within tumors. The effect of TKI treatment on MRI was predominantly in the tumor center, whereas the tumor periphery maintained its initial features (ie, high BV and low TP) during treatment. Such regional differences in the effect of antiangiogenic treatment have been introduced in several studies.^32^, ^33^, ^34^ One explanation for these treatment effects in the tumor center may be “vascular normalization,” which suppresses “vascular regression” by restoring the balance between pro‐and antiangiogenic factors, thereby converting abnormal tumor vessels to a more normal state.^32^, ^35^ More specifically, vascular normalization processes “prune” immature vessels and improve the solidity of the remaining vasculature by enhancing the coverage of perivascular cell and basement membrane.^36^ This effect was demonstrated as increased NG2 expression (ie, increased pericyte coverage) and decreased α‐caspase 3 expression (ie, suppressed apoptosis) in our histologic examination. As a supportive evidence, Dominietto et al^37^ recently demonstrated that anti‐VEGF treatment led to a reduced number of small vessels and apparently less chaotic organization of the vascular network. In addition to a vascular normalization effect, treatment‐induced vessel disintegration and tumor apoptosis have also been proposed as factors that decrease TP in the tumor center.^33^ In fact, Obad et al^34^ suggested that normalized vascular morphology may not improve vascular function and eventually results in poor tumoral blood flow and increased hypoxia.

Interestingly, compared with the tumor center, the tumor periphery exhibited no statistically meaningful treatment‐induced change on MRI. Although our observations may depend on intrinsic characteristics of C6 glioma and sorafenib, spatially heterogeneous treatment response was also noted in several previous studies. For example, the tumor periphery exhibited maintenance of high BV and vascular integrity against monoclonal antibody to VEGF/VEGFR or vessel‐targeting treatment.^38^, ^39^, ^40^ Therefore, preservation of inherent vascular phenotypes against antiangiogenic treatment in the tumor periphery can be regarded as a process in the tumor that potentiates drug resistance. The resistance to vessel‐targeting agents has been intensively investigated in studies with vascular disrupting agents. In those studies, despite extensive necrosis in the tumor center, a viable rim of tumor cells survived in the tumor periphery, which subsequently led to regrowth.^38^, ^41^, ^42^ As an important mechanism of remaining viable rim, it has been discovered that vascular stabilization by high pericyte coverage in the tumor periphery provides inherent resistance to vascular disrupting agents. As mentioned, vessel generation and stabilization in the tumor periphery is reflected by high BV and low TP on MRI. Therefore, our MRI findings may be used as biomarkers to predict and monitor the tumor response to antiangiogenic treatment in preclinical studies.

In addition to the inherent potential against vessel‐targeting agents, including high pericyte coverage of vessels, acquired drug resistance, such as over‐expression of alternative signaling pathways, is recognized as an important mechanism of tumor evasion from angiogenic suppression.^43^, ^44^ In this study, whereas the expression of VEGFR2, a major target of sorafenib, diminished in both the tumor center and periphery, the activities of CD31 (vessel formation) and Ki67 (cell proliferation) were preserved in the tumor periphery. This different response to treatment between VEGFR2 and CD31/Ki67 may be closely associated with acquired resistance to TKI treatment. We suggest that biologic processes other than VEGF‐mediated angiogenesis may elicit the maintenance of angiogenesis to supply tumor growth in the tumor periphery.

This study applied dual injection of extravascular fluid (ie, Gd‐DOTA) and intravascular (SPION) CAs to separately measure BV and TP, instead of simultaneously calculating TP and BV from a PK model‐based analysis of Gd‐DOTA using DCE‐MRI. The main rationale of our approach was to avoid unreliability and inaccuracy of the PK‐model‐based method. It has been established that wide variability in estimating arterial input function leads to substantial deviations in calculating TP and BV parameters.^45^, ^46^ Moreover, PK modeling is significantly influenced by B_1_ field inhomogeneity. 47 According to a recent simulation,^47^ pharmacokinetic parameters (ie, K^trans^ and v_p_) fluctuate up to eight times wider than non‐model‐based parameters (such as MaxEnh and IAUC) under the same B_1_ inhomogeneity. Therefore, based on validated reliability in our previous study,^17^ we separately assessed the TP parameter from a non‐model‐based approach of DCE‐MRI and BV parameter from SPION‐induced ΔR_2_(*).

This study analyzed vascular phenotypes of orthotopic C6 glioma, which typically exhibited high TP in the tumor center and high BV in the tumor periphery. However, MRI features of tumor vasculature may vary according to numerous factors, including tumor cell properties, timing of evaluation, and method of tumor generation. For example, contrary to our results, brain metastasis models of Mel57‐VEGF‐A cells demonstrated only slight SPION‐induced R_2_* decrease but no Gd leakage at an early stage.^48^ Therefore, the angiogenic alterations observed in this study cannot be generally extended to all solid tumors. Nevertheless, this study provides a rationale for spatiotemporal vascular evaluation using both intravascular and extracellular fluid CAs. Regardless of the variety of angiogenic presentations, TP and BV are the main parameters to describe tumor angiogenesis, and their spatiotemporal manifestation can adequately reflect treatment‐induced alterations of tumor vascular phenotypes.

As another potential limitation, the region‐oriented analysis might be affected by rapid tumor growth, particularly between days 4 and 7. Indeed, paired comparison between time points may be incorrect because the former tumor periphery became tumor center on the follow‐up MR images. Nevertheless, the temporal changes of center‐to‐periphery ratio of vascular parameters clearly present the time‐dependent alteration in distribution of vascular characteristics.

In summary, this study quantitatively demonstrated intratumoral heterogeneity in the distribution of TP and BV parameters. This regional heterogeneity may be closely related to spatiotemporal variations in antiangiogenic treatment effects. Tumor centers exhibited apparent treatment‐induced vascular normalization; however, the tumor periphery maintained its inherent vascular features against TKI treatment. Given the necessities to understand the mechanism of tumor resistance to antiangiogenic treatment, quantitative assessment of spatiotemporal heterogeneity in tumor vascular phenotypes may provide important information regarding tumor responses to therapeutic agents.

## CONFLICT OF INTEREST

None declared.
